# Protein characterization, purification, and sequence analysis data for plant-made catfish interleukin 22

**DOI:** 10.1016/j.dib.2020.106637

**Published:** 2020-12-21

**Authors:** Lana Elkins, Maureen C. Dolan

**Affiliations:** a Molecular Biosciences Program, United States; b Arkansas Biosciences Institute, United States; c Department of Biological Sciences, Arkansas State University, P.O. Box 639, Jonesboro, AR 72467, United States

**Keywords:** Interleukin 22, Therapeutant, Recombinant protein, Plant-based production

## Abstract

Production and purification of a novel protein in plants results in the generation of multiple data sets leading to an optimized protocol for recovering the recombinant protein. This article presents the data collected in the process used to produce, purify and validate a catfish interleukin 22 (cfIL-22) expressed using a plant-based platform. A commonly used workflow for confirming optimal expression and extraction of the recombinant protein was employed and is outlined herein. The complete research article, including activity analysis of plant-produced cfIL-22, is published in Journal of Biotechnology Elkins and Dolan [1]. Data collected in optimizing the expression, purification and characterization process of cfIL-22 includes stained protein gels and western immunoblot analyses, DNA and protein sequencing, post-translational modification predictions and protein structure predictions. The value of this data lies not only in future work in expressing interleukin 22 orthologs but also provides a guide for optimizing the production and validating similar complex animal/human proteins produced in plants.

## Specifications Table

**Table:** 

Subject	Biochemistry, genetics, and molecular biology
Specific subject area	Production of a recombinant immune protein in plants for use in veterinary medicine
Type of data	Image Figure
How data were acquired	Channel catfish Interleukin 22 (cfIL-22) coding sequence was provided by a colleague, Dr. Sylvie Quiniou (USDA-ARS-Catfish Genetics Research Unit, Stoneville MS; Accession#MK956102). All plant leaf-derived samples were resolved by SDS-PAGE and detected by western immunoblotting with anti-histidine antibody and visualized using chemiluminesence with film for monitoring expression of cfIL-22 with a 6X histidine tag; post translational modification and protein sequence analyses were predicted using freeware on Expasy.org and MS/MS analysis; protein structure prediction was obtained using I-TASSER and Raptor X modeling software. Instruments: film developer Make and model and of the instruments used: SRX-101A Konica Minolta
Data format	Raw Analyzed
Parameters for data collection	Parameters considered for plant infiltration included age of plant (4 weeks post germination) and concentration of the Agrobacteria infiltrate (0.2 OD_600_). Western immunoblot analyses parameters to consider is equivalent sample loading was normalized based on total volume
Description of data collection	Recovery of total soluble protein extracted from infiltrated leaf tissue using a mortar and pestle was collected for western immunoblot data. Sequence and structure data were collected using software mentioned previously.
Data source location	Institution: Arkansas Biosciences Institute, Arkansas State University City/Town/Region: Jonesboro, AR Country: USA
Data accessibility	With the article Repository name: Mendeley Data https://doi.org/10.17632/t2tkykvwb4.2
Related research article	Elkins, Lana and Dolan, Maureen C. Plant Production and Functional Characterization of Catfish Interleukin-22 as a Natural Immune Stimulant for Aquaculture Fish. Journal of Biotechnology. In Press. https://doi.org/10.1016/j.jbiotec.2020.10.017

## Value of the Data

• These data are useful in providing important experiments to include when characterizing a novel recombinant protein and determining optimal conditions for its expression in plants.
• The most direct beneficiaries of this data would be scientists interested in recombinant expression of catfish interleukin 22. These data would also provide insights for developing a workflow to express and produce interleukin 22 protein in other heterologous expression systems. Most broadly, this data is important to consider, when generating a QA/QC checklist, for producing any novel protein using a whole plant production platform.
• The data can be used to identify optimal expression kinetics of similar recombinant proteins produced in plants and other eukaryotic host systems, various useful extraction buffers for improved protein recovery and freely accessible bioinformatics software. This data can be used in generating a systematic and robust roadmap for optimizing production of other recombinant proteins in plants.

## 1 Data Description

**Fig. 1:**
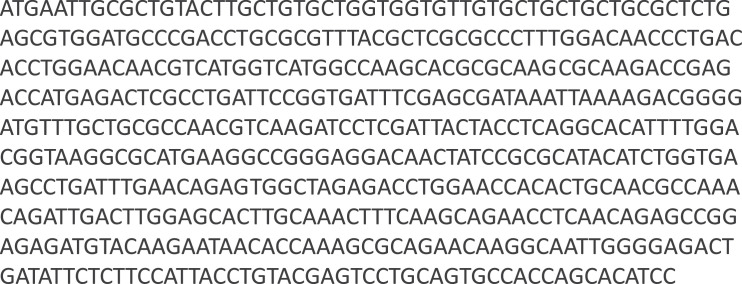
Channel catfish Interleukin 22 coding sequence.

**Fig. 2:**
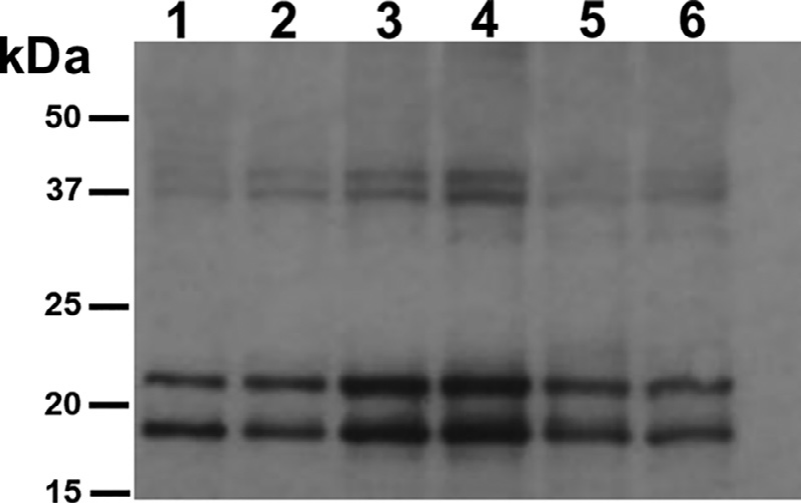
*cfIL-22 expression kinetics in tobacco leaf tissues.* cfIL-22 was expressed using the plant **t**ransient expression platform. Crude protein extracts from leaf tissue collected at 48, 72, 96, 120, 144, and 168 h post infiltration, (samples 1–6 respectively) were resolved by SDS-PAGE and detected using an anti-His-western blot. Optimal time point for collection of tissue for production of monomeric forms of recombinant cfIL-22 was 96 h post-infiltration.

The channel catfish interleukin 22 (cfIL-22) accession **#MK956102** 531 nucleotides ([2]; Fig. 1) and 177 amino acids ([2]; Fig. 2) sequences are presented. The amino acid sequence is annotated to highlight sequence features including: signal peptide, phosphorylation sites, and N-glycosylation site. The signal peptide was initially predicted using SignalP prediction software and confirmed with N-terminal sequencing of the plant-produced and purified cfIL-22 protein. The phosphorylation and N-glycan sites were predicted using softwares on Expasy.org. N-glycosylation of the recombinant protein was confirmed using a standard deglycosylation with PNGase F. The amino acid sequence of the purified recombinant cfIL- 22 was validated using mass spectrometry (MS/MS analysis).

**Fig. 3:**
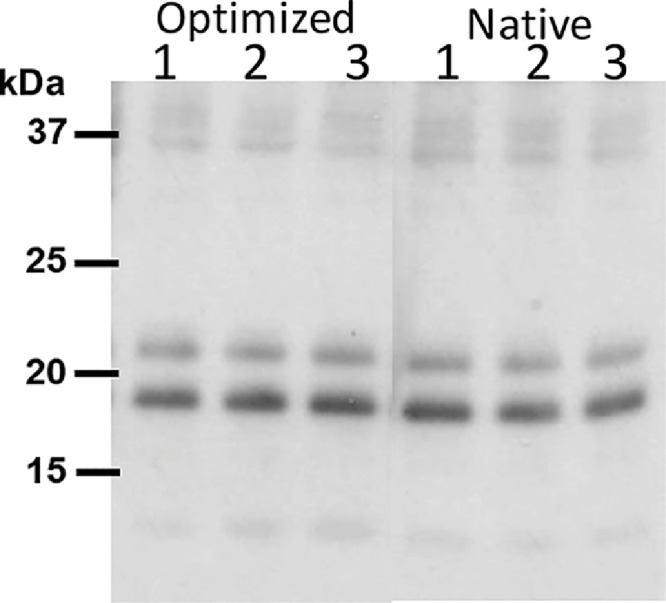
*Comparison of native and tobacco codon optimized cfIL-22 expression. A. tumefaciens* transformed with **g**ene constructs coding for tobacco codon optimized and native cfIL-22 were vacuum infiltrated into 3 independent tobacco plants. Crude protein extracts of leaf tissue were resolved on a 12% SDS-PAG and detected with α-His-antibody. No significant difference in the monomeric cfIL-22 bands were observed.

For detecting the channel catfish interleukin 22 (cfIL-22) with a genetically fused, carboxy-terminus, 6X histidine tag, a western immunoblot using an anti-histidine antibody was performed ([2]; Fig. 3). Protein samples for this analysis were generated from plant leaf tissue, infiltrated with the cfIL-22 plant expression construct. Leaf tissues were collected every 24 h post-infiltration starting at 48 h through 168 h. The bands at ∼19 kDa and 22 kDa correspond to a predicted cfIL-22 monomer and bands at ∼38 kDa and 44 kDa correspond to a cfIL-22 dimer. The relative intensity of the protein bands on anti-his western immunoblots was used in selecting the optimal post-infiltration leaf collection time. Protein bands extracted from SDS-PAGs were used in confirming protein identity, by MS/MS analysis, with 82% protein coverage.

**Fig. 4:**
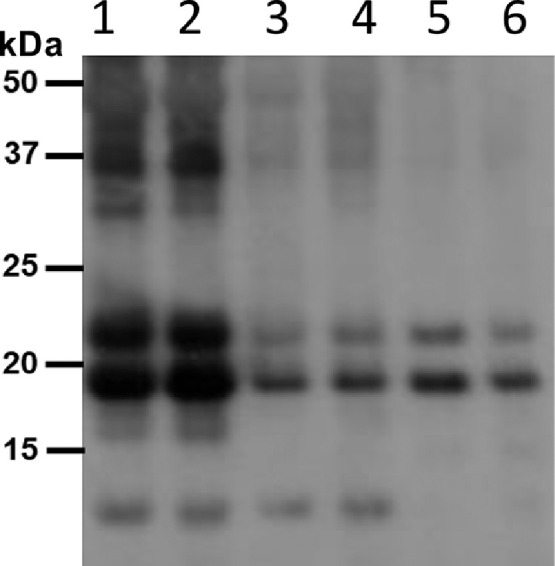
*Protein extraction buffer determination.* Three buffers across a wide range of pH's were tested to find optimal extraction of cfIL-22 from plant leaves. Tris pH 8.5 (1,2 -/+ NaCl), phosphate buffer pH 7.2 (3,4 -/+ NaCl) and citrate buffer pH 6.5 (5,6 -/+ NaCl) were tested. CfIL-22 pI is 8 so it was suspected that the higher pH buffer would help with extraction. Each buffer was also tested +/- salt. The Tris pH 8.5 buffer was the best at extracting with no benefit to addition of salt. Crude protein extracts resolved on a 12% SDS-PAGE and detected by α-His-antibody.

An anti-His-western immunoblot was used to compare relative expression levels of two different expression constructs for cfIL-22 in plants ([2]; Fig. 4). Optimized corresponds to protein generated by codon usage aligned with optimal protein expression in tobacco plants. The recombinant cfIL-22 was also expressed using the native coding sequence in catfish.

**Fig. 5:**
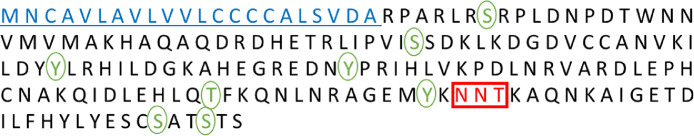
*Post translational modifications of cfIL-22*. The signal peptide was predicted using SignalP prediction software and was later confirmed by N-terminal sequencing. Highlighted in red is a possible N-glycosylation site although using NetNglyc predicted this site would not be glycosylated. In green circles are the possible phosphorylation sites predicted by NetPhos. All software used was located on Expasy.org. MS/MS analysis was carried out on the three bands resolved using SDS PAGE. Protein was transferred to PVDF membrane and stained with Coomassie blue safe stain. Bands were cut out and sent for sequencing. Sequencing confirms all three bands are cfIL-22 with 82% protein coverage.

To identify the best protein extraction conditions for maximizing recovery of cfIL-22 from the leaf tissues, three different buffers were tested. Using isoelectric point (pI) prediction software (Geneious 8.0.4), cfIL-22 has a predicted pI of 7.5 supporting use of a higher pH buffer to favor its extraction. Extraction of cfIL-22 was compared using a basic, neutral or acidic buffer and analysed by anti-his western immunoblotting ([2]; Fig. 5).

**Fig. 6:**
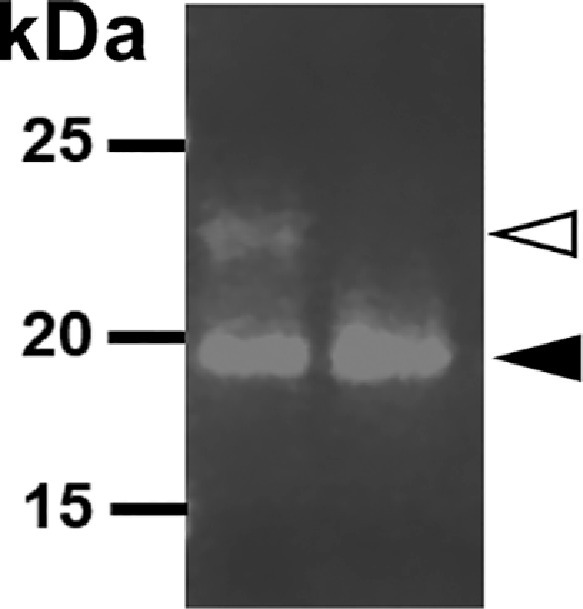
Upper band recognized as cfIL-22 is N-glycan form. Treatment with PNGaseF enzyme showed removal of upper band and presence on of the lower band shown in lane 2 (black arrow). This indicates the upper band (white arrow) is an N-glycan form of cfIL-22.

To confirm the slower migrating 22 kDa band on western immunoblots was a glycan variant of the predicted cfIL-22 monomer (19 kDa)([2]; Fig. 3-5), a deglycosylation assay was performed. Purified cfIL-22 protein was treated with PNGaseF; an amidase which cleaves the bond between an N-glycan and the asparagine residue within the primary sequence of the protein. Reactions were resolved on a Coomassie-stained SDS-PAG ([2]; Fig. 6). The upper band disappears in the enzyme-treated lane relative to the 22 kDa band in the untreated sample. In addition, an increased intensity of the lower unglycosylated band (19 kDa) in the treated sample relative to untreated sample is visible.

**Fig. 7:**
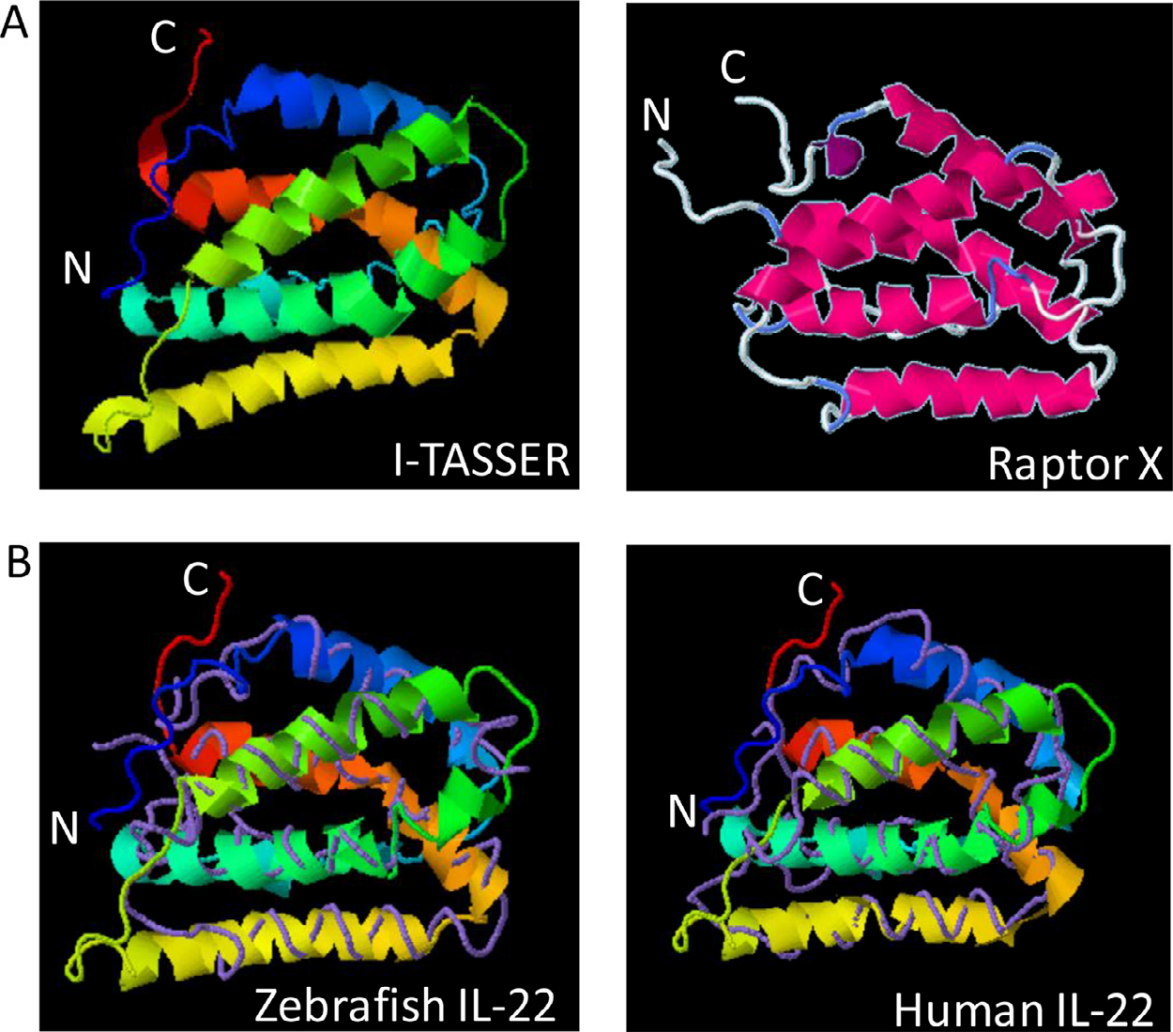
Predicted structure of catfish IL-22. The structure prediction software I-TASSER and Raptor X were used to predict the 3-D structure of cfIL-22 (A). The I-TASSER model (ribbon structure) was overlayed with zebrafish IL-22 and human IL-22 (purple backbone structure, B).

The predicted 3-D structure of cfIL-22 using two different prediction softwares, I-TASSER and RaptorX, is shown ([2]; Fig. 7A) respectively. I-TASSER predicted model ribbon structure of cfIL-22 was overlaid on the backbone model of zebrafish IL-22 with strong alignment ([2]; Fig. 7B, panel 1). Of the published IL-22 sequences in the NCBI database at the time of analysis (December 2018), cfIL-22 closely aligns to the zebrafish orthologue. Human IL-22 is the most well characterized IL-22 orthologue in both structure and function. I-TASSER cfIL-22 ribbon model overlaid with the human IL-22 crystal structure also shows strong alignment ([2]; Fig. 7B, Panel 2).

## 2 Experimental Design, Materials and Methods

### 2.1 cfIL-22 cloning and expression construct design

Catfish IL-22 nucleotide sequence was provided by Dr. Sylvie Quiniou (USDA-ARS-Catfish Genetics Research Unit, Stoneville MS; Accession **#MK956102**). Synthetic DNA with genetic sequences corresponding to the native catfish IL-22 sequence (cfIL-22) and a codon-optimized sequence for expression in *N. benthamiana* (cfIL-22 opt) were synthesized (GeneArt®, Thermo Fisher Scientific, Waltham, MA). For both variants, the mature coding sequence of cfIL-22 gene was genetically fused with a 5′ native signal peptide and a 3′ 6X-histidine tag. Both variants of cfIL-22 were cloned into an ampicillin resistant vector (pMA) and sequences verified by the manufacturer.

Gene cassettes were cloned into a pBIB-Kan plant expression vector [3] downstream of the constitutive dual-enhanced 35S Cauliflower Mosaic Virus (deCaMV) promoter (35S) [4], and a translational enhancer from the tobacco etch virus (TEV) [5] and upstream of the Tnos terminator. Plasmids were transformed into Top 10β *E. coli,* screened by PCR, and gene sequences confirmed by Sanger sequencing (CRC DNA Sequencing Facility, University of Chicago).

### 2.2 Recombinant protein expression using an *agrobacterium*-mediated transient plant production system

Expression constructs of the two variants of the cfIL-22 gene were transformed into *Agrobacterium tumefaciens* strain LBA4404 using the freeze/thaw method [6]. Following plasmid prep, PCR screening and confirmation by Sanger sequencing, lead *A. tumefaciens* (*Agro*) lines with cfIL-22 expression constructs were preserved as glycerol stocks (30% glycerol) and stored at −70 °C.

Prior to transient expression in plants, ultracold-preserved, *Agro* cfIL-22 culture lines were plated and incubated at 28 °C on YEP agar plates [10 g/L Bacto-peptone (Difco), 10 g/L yeast extract (Difco), 5 g/L NaCl (Sigma-Aldrich), pH 7.0] containing 0.1 g/L of kanamycin (Sigma-Aldrich; antibiotic selection of the expression construct) and 0.06 g/L of streptomycin (Sigma-Aldrich; selection of the *Agro* binary vector). Note, plated *Agro* constructs used for inoculating liquid cultures were used within 2 weeks of plating.

Two to three colonies of a given clone were inoculated into 5 ml YEP medium and cultured at 28 °C, on an orbital shaker, at 225 rpm, for ∼2 days. The 5 ml bacterial suspensions were transferred to 50 ml of YEP medium containing the antibiotics and cultured for an additional 24 h. To obtain accurate and reproducible quantification of the *Agro* suspensions, an aliquot of the 50 ml *Agro* culture was diluted 1:10 in YEP and gentle inversion used to obtain a homogenously mixture prior to measuring the optical density (OD) at 600 nm wavelength by spectrophotometry (BioRad) in a 1 ml disposable cuvette. *Agro* cultures were then diluted into induction medium (IM; 10 mM MES pH 5.5) at an OD_600_ of 0.2 immediately prior to infiltrating into *Nicotiana benthamiana* plants.

Transient expression in *Nicotiana benthamiana* plants was carried out using an *Agrobacterium*-mediated vacuum infiltration method (*Agro*-infiltration) previously described [7]. Briefly, 4–6-week-old *N. benthamiana* plants were grown and maintained under controlled temperature (25 °C for 16 hrs light cycle/ 21 °C for 8 hrs dark cycle), light intensity (150 μmol) and humidity (70% RH). Plants were vacuum infiltrated with *Agro* cultures containing the cfIL-22 gene. A 500 ml *Agro* culture is sufficient to infiltrate approximately up to 16 plants for each expression construct. An *Agro* culture containing an empty pBK vector was used in generating plant tissue for the protein negative control.

*Agro*-infiltrated tobacco plants were returned to the environmental growth chamber. For determining the optimal harvest time, leaf tissues were collected daily from a single plant beginning at day 2 (48 h) through day 7 (168 h) post-infiltration. At each collection time, all leaf tissue was, weighed, transferred to storage bags, frozen in liquid nitrogen and stored at −70 °C until further analysis. After optimal expression of cfIL-22 in this host system was established, plant tissues were similarly collected and stored in 100 g aliquots for later cfIL-22 purification.

### 2.3 Recombinant protein extraction and characterization

For initial studies, 0.5 g frozen leaf tissues were weighed and ground using a mortar and pestle. As frozen leaf tissue collection ranged from 5 to 100 g aliquots, to ensure representative sampling, the frozen tissue was hand crushed and thoroughly mixed prior to removing the 0.5 g sample. Extraction buffer containing 2% PVPP (100 mM Tris pH 8.5; 100 mM citrate pH 6.5; 100 mM phosphate buffer pH7.2) was added and further ground to a chilled liquid slurry. Extracts were transferred to 2 mL microfuge tubes and clarified by centrifugation at 16,000x*g*, for 30 min, at 4 °C. Supernatants were collected and samples were prepared for resolution on reducing, SDS-PAGE. Protein supernants were prepared at a final concentration of 1X loading dye solution (LDS; Thermo Scientific) and 0.1 M DTT, subject to denaturation at 100 °C for 10 min. Qualitative and quantitative assessment of the protein included SDS-PAGE (NuPage 12% Bis-Tris; Invitrogen) resolution and detected either by western immunoblotting using an anti-His-monoclonal antibody (Genscript) and chemiluminescent detection (SuperSignal West Pico PLUS) or directly staining with Coomassie Simply Blue Safe Staining (Invitrogen).

For MS/MS analysis ∼ 1ug of the purified recombinant cfIL-22 (purification details in accompanying article, Elkins and Dolan, [1]) was resolved on SDS-PAGE (Invitrogen, Novex, NuPage, 12% BisTris) and detected with Coomassie Blue Simply Safe stain. The bands corresponding to cfIL-22 were excised and sent to the University of Arkansas for Medical Sciences (UAMS) Proteomics Core Facility for analysis.

Protein preparation for N-terminal sequencing involved resolving purified cfIL-22 by SDS-PAGE and electrotransfer to PVDF membrane. Following Coomassie staining (Simply Blue Safe stain), the membrane was destained using 20% methanol. The visible bands on the membrane were excised using a sterile scapel blade and sent to the Protein Facility (Office of Biotechnology, Iowa State University).

Digestion of N-glycans from purified cfIL-22 protein was performed using the PNGase F enzyme (New England Biolabs) in accordance withmanufacturer’s guidelines. Breifly, 1 μg cfIL-22 purified protein samples were digested with PNGase using the denauring protocol, for 1 hr, at 37 °C. The glycodigests were comparatively analyzed to undigested cfIL-22 samples (mock control) immediately following the reaction period by SDS-PAGE and western immunoblotting.

For structural predicitons of cfIL-22 protin, amino acid sequences (published and experimentally established in this study) were was submitted both to I-TASSER and Raptor X modeling programs.

## Ethics Statement

Not required.

## CRediT Author Statement

**Lana Elkins**: Conceptualization, Data curation, Formal analysis, Investigation, Methodology, Validation, Visualization, Writing- original draft. **Maureen C. Dolan**: Conceptualization, Funding acquisition, Investigation, Methodology, Project administration, Resources, Supervision, Validation, Writing- review and editing.

## Declaration of Competing Interest

The authors declare that they have no known competing financial interests or personal relationships that have, or could be perceived to have, influenced the work reported in this article.
